# Supplementary material for: Climate teleconnections modulate global burned area
Source: Nat Commun. 2023 Jan 26;14:427. doi: 10.1038/s41467-023-36052-8 (PMC9879971; doi:10.1038/s41467-023-36052-8)
Supplement: Supplementary file 1 — Supplementary Information [file 41467_2023_36052_MOESM1_ESM.docx]

**Supplementary Information File**

**Climate teleconnections modulate global burned area**

Cardil Adrián^1,2,3,*^, Rodrigues Marcos^4,5^, Tapia Mario^2^, Barbero Renaud^6^, Ramírez Joaquin^2^, Stoof CathelijneR^7^, Silva Carlos Alberto^8^, Mohan Midhun^9^, de-Miguel Sergio^1,3,*^

*Corresponding authors: Adrián Cardil ([acardil@tecnosylva.com](mailto:acardil@tecnosylva.com)); Sergio de-Miguel (sergio.demiguel@udl.cat)

**Supplementary correlation analysis**

To analyze the linkages between CTs and BA, both the Spearman’s and Pearson’s R correlation coefficients were calculated between seasonal time series of BA during the fire season peak and CTs at pixel level (0.5ºx0.5º resolution). These statistical analyses consider the correlation between two sets of data (CTs and BA). The Spearman's rank-order correlation is the nonparametric version of the Pearson product-moment correlation. Spearman's correlation coefficient measures the strength and direction of the monotonic association between the two studied variables. Both statistical analyses identified similar associations and spatial patterns between BA and all CTs for all studied lags. The spatial pattern of correlations for both statistical approaches are summarized in Fig. S1, S2, S3, S2, S3, S4, S5, S6, S7 and S8, depicting the direction (either positive or negative) and the significance (p<0.10 and p<0.05) of the CT-fire association.


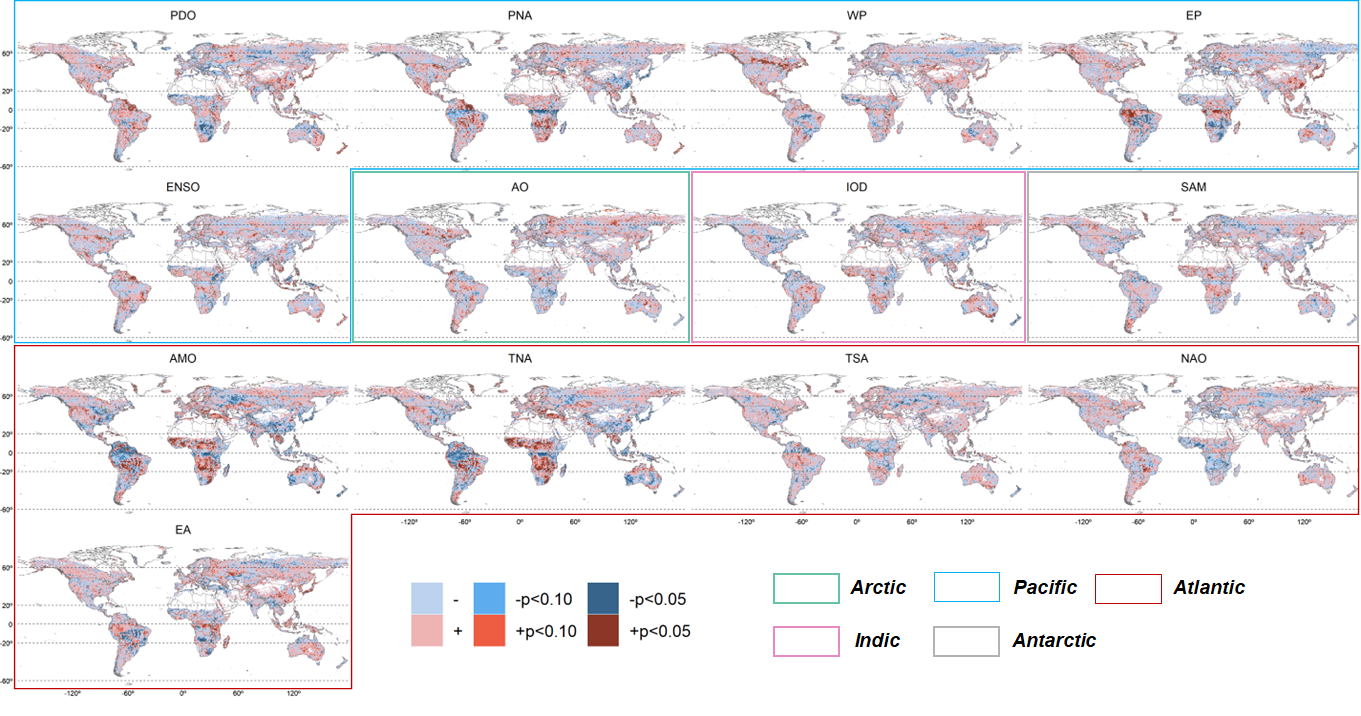


**Figure S1. Synchronous (0-month lag) Pearson R correlation between climate teleconnections (CT) and burned area (BA) during the fire season peak from 1982 to 2018.** The maps at 0.5º pixel resolution represent the correlations at P<0.05 (|R| > 0.34) and P<0.10 (|R| > 0.29) between BA and the studied CTs distributed throughout the globe (Arctic, Pacific, Atlantic, Indic and Antarctic) described in the methods section S.1.1. Atlantic Multidecadal Oscillation (AMO); Arctic Oscillation (AO); East Atlantic (EA); El Niño 3.4 SST index (ENSO); Eastern Pacific (EP); Indian Ocean Dipole (IOD); North Atlantic Oscillation (NAO); Pacific Decadal Oscillation (PDO); Pacific North American (PNA); Southern Annular Mode (SAM); Tropical North Atlantic (TNA); Tropical South Atlantic (TSA); Western Pacific (WP)


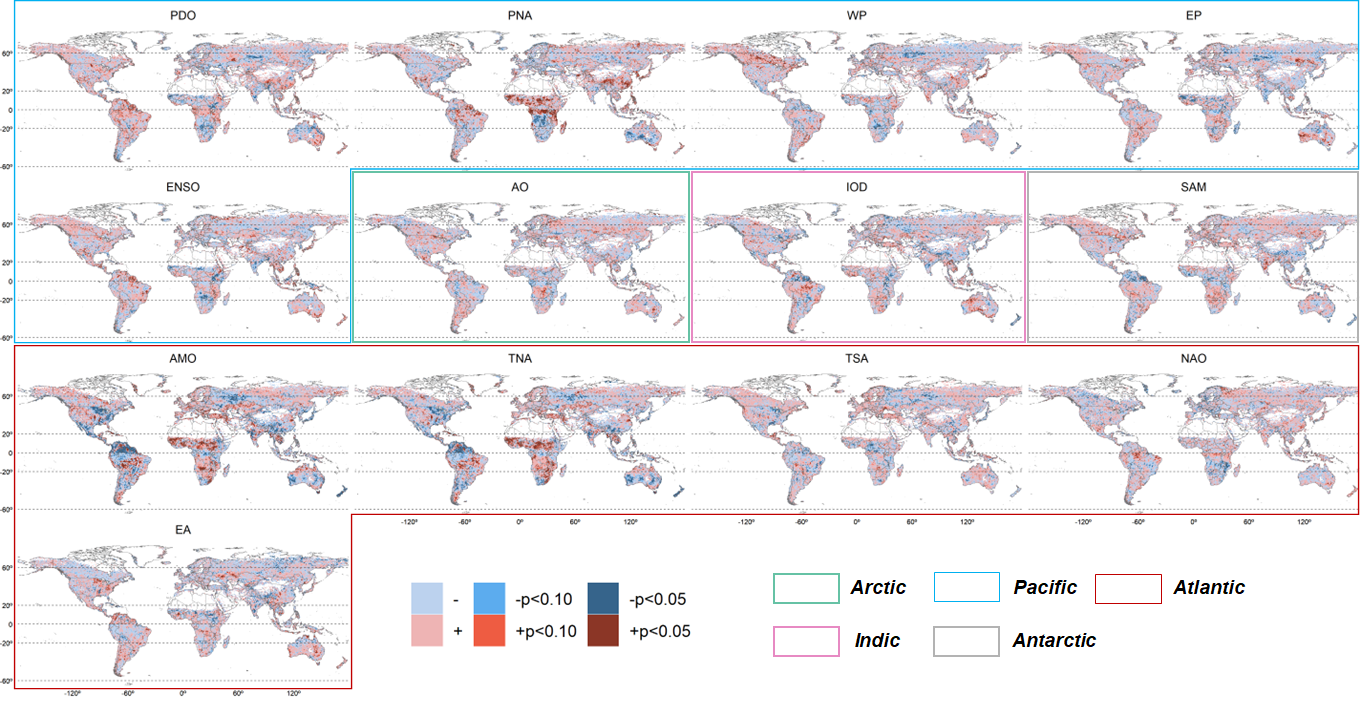


**Figure S2. Lagged (3-month lag) Pearson R correlation between climate teleconnections (CT) and burned area (BA) during the fire season peak from 1982 to 2018.** The maps at 0.5º pixel resolution represent the correlations at P<0.05 (|R| >= 0.34) and P<0.10 (|R| >= 0.29) between BA and the studied CTs distributed throughout the globe (Arctic, Pacific, Atlantic, Indic and Antarctic) described in the methods section S.1.1. Atlantic Multidecadal Oscillation (AMO); Arctic Oscillation (AO); East Atlantic (EA); El Niño 3.4 SST index (ENSO); Eastern Pacific (EP); Indian Ocean Dipole (IOD); North Atlantic Oscillation (NAO); Pacific Decadal Oscillation (PDO); Pacific North American (PNA); Southern Annular Mode (SAM); Tropical North Atlantic (TNA); Tropical South Atlantic (TSA); Western Pacific (WP)


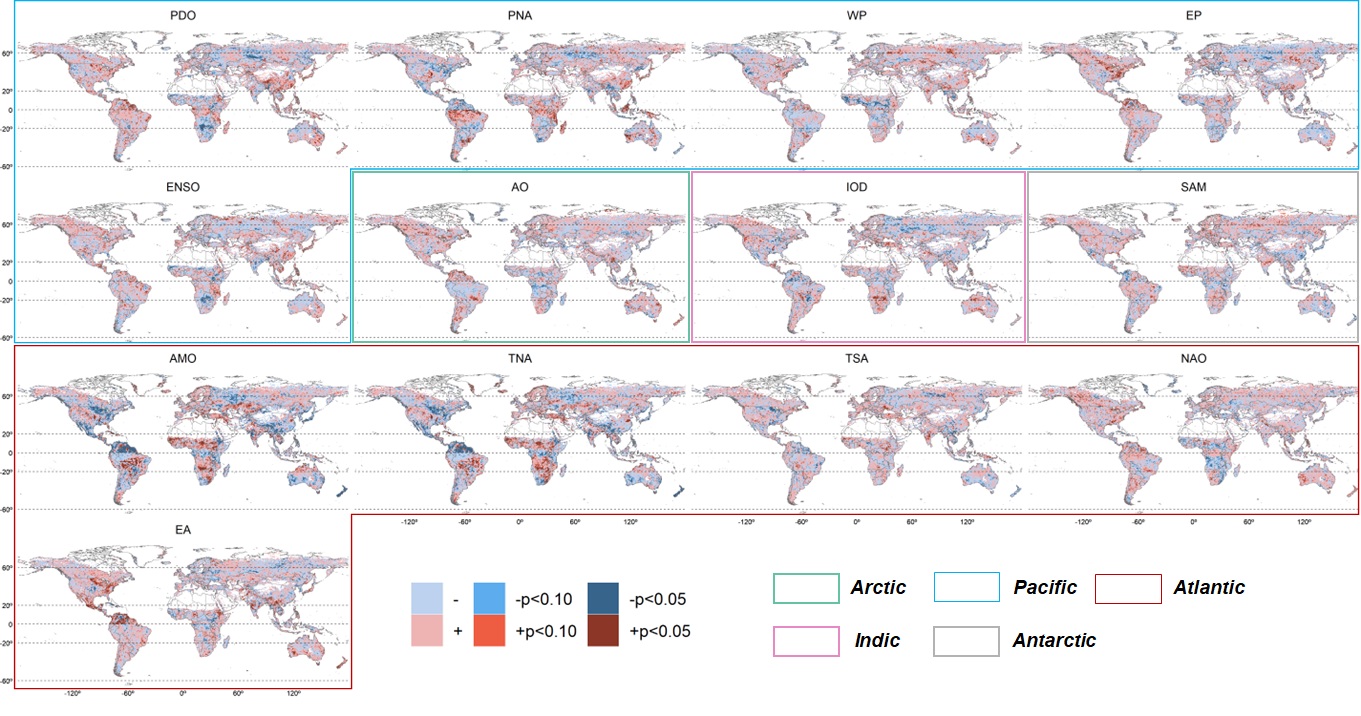


**Figure S3. Lagged (6-month lag) Pearson R correlation between climate teleconnections (CT) and burned area (BA) during the fire season peak from 1982 to 2018.** The maps at 0.5º pixel resolution represent the correlations at P<0.05 (|R| >= 0.34) and P<0.10 (|R| >= 0.29) between BA and the studied CTs distributed throughout the globe (Arctic, Pacific, Atlantic, Indic and Antarctic) described in the methods section S.1.1. Atlantic Multidecadal Oscillation (AMO); Arctic Oscillation (AO); East Atlantic (EA); El Niño 3.4 SST index (ENSO); Eastern Pacific (EP); Indian Ocean Dipole (IOD); North Atlantic Oscillation (NAO); Pacific Decadal Oscillation (PDO); Pacific North American (PNA); Southern Annular Mode (SAM); Tropical North Atlantic (TNA); Tropical South Atlantic (TSA); Western Pacific (WP)


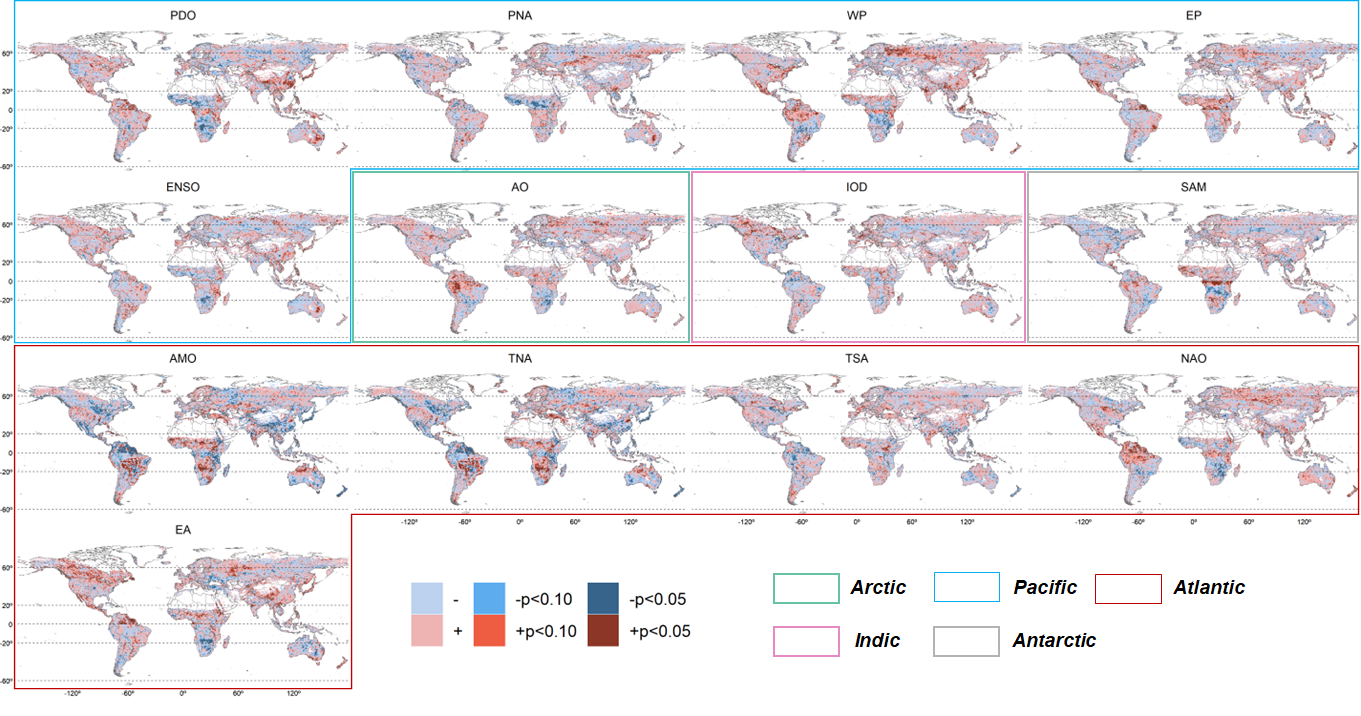


**Figure S4. Lagged (9-month lag) Pearson R correlation between climate teleconnections (CT) and burned area (BA) during the fire season peak from 1982 to 2018.** The maps at 0.5º pixel resolution represent the correlations at P<0.05 (|R| >= 0.34) and P<0.10 (|R| >= 0.29) between BA and the studied CTs distributed throughout the globe (Arctic, Pacific, Atlantic, Indic and Antarctic) described in the methods section S.1.1. Atlantic Multidecadal Oscillation (AMO); Arctic Oscillation (AO); East Atlantic (EA); El Niño 3.4 SST index (ENSO); Eastern Pacific (EP); Indian Ocean Dipole (IOD); North Atlantic Oscillation (NAO); Pacific Decadal Oscillation (PDO); Pacific North American (PNA); Southern Annular Mode (SAM); Tropical North Atlantic (TNA); Tropical South Atlantic (TSA); Western Pacific (WP)


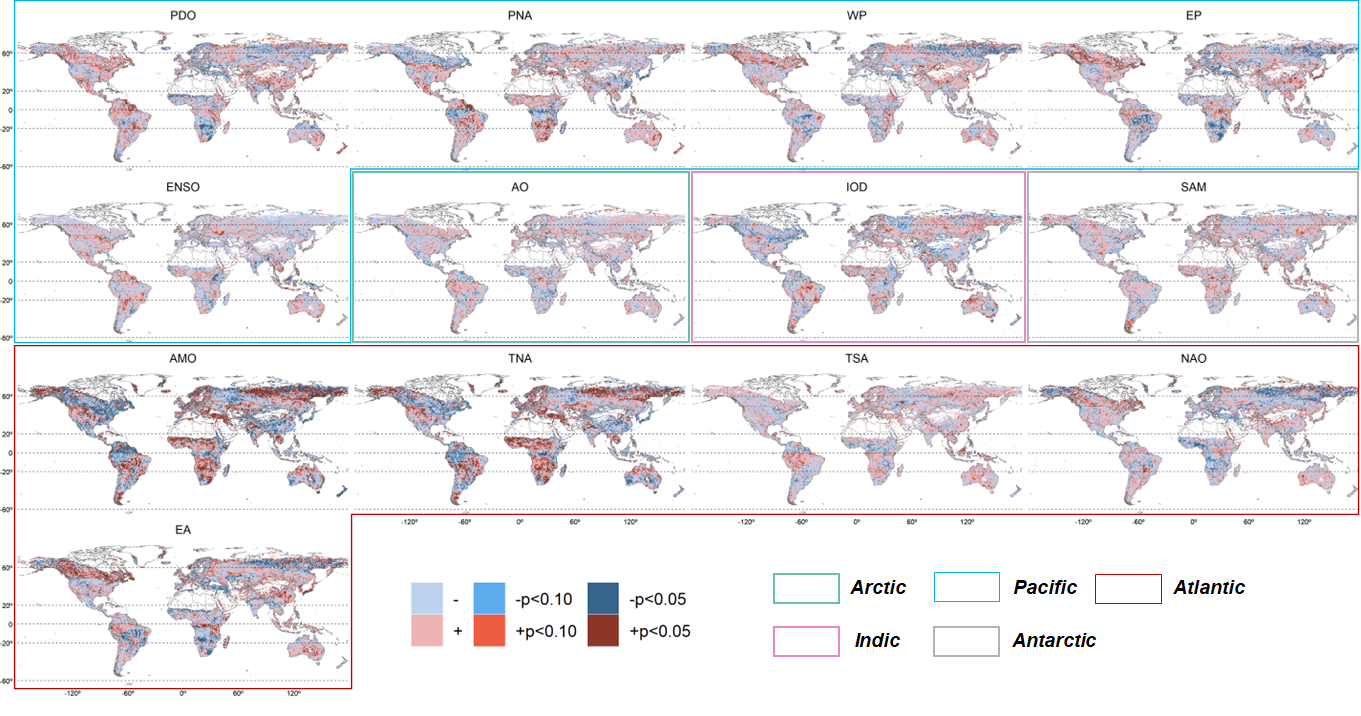


**Figure S5. Synchronous (0-month lag) Spearman *rho* correlation between climate teleconnections (CT) and burned area (BA) during the fire season peak from 1982 to 2018.** The maps at 0.5º pixel resolution represent the correlations at P<0.05 (|R| >= 0.34) and P<0.10 (|R| >= 0.29) between BA and the studied CTs distributed throughout the globe (Arctic, Pacific, Atlantic, Indic and Antarctic) described in the methods section S.1.1. Atlantic Multidecadal Oscillation (AMO); Arctic Oscillation (AO); East Atlantic (EA); El Niño 3.4 SST index (ENSO); Eastern Pacific (EP); Indian Ocean Dipole (IOD); North Atlantic Oscillation (NAO); Pacific Decadal Oscillation (PDO); Pacific North American (PNA); Southern Annular Mode (SAM); Tropical North Atlantic (TNA); Tropical South Atlantic (TSA); Western Pacific (WP)


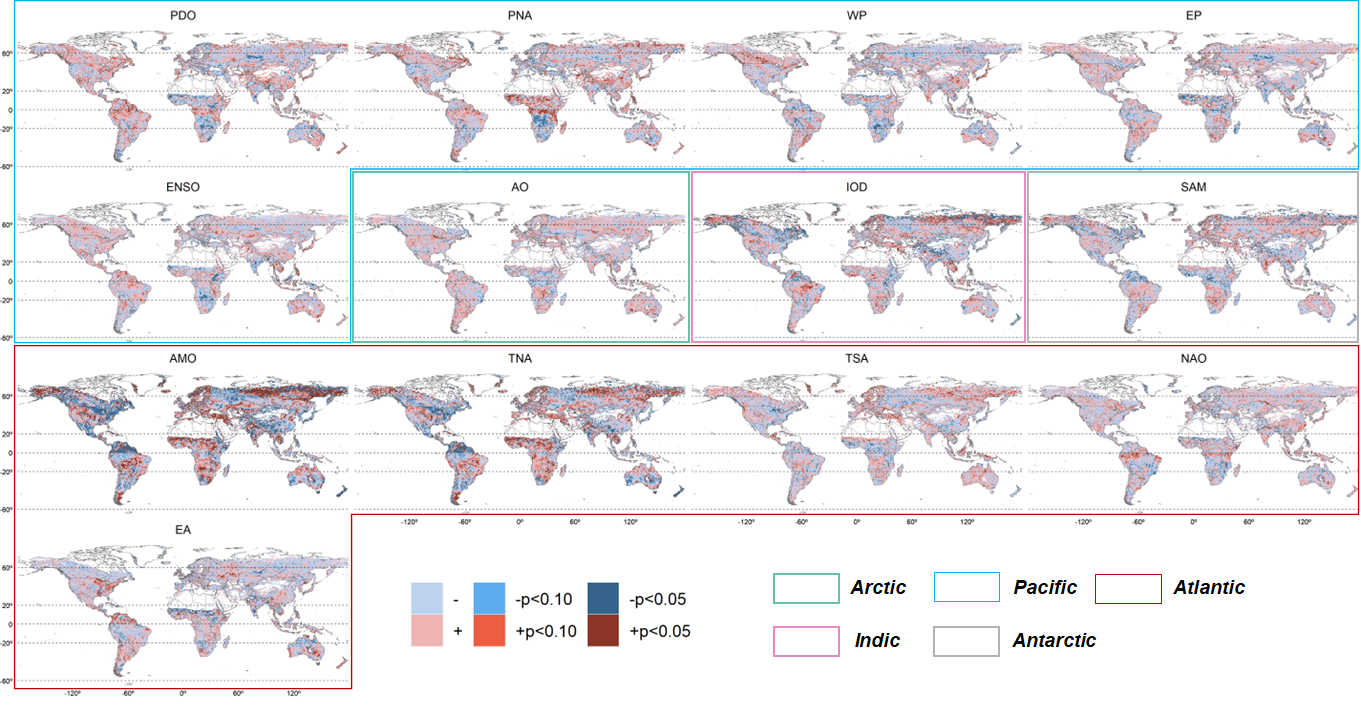


**Figure S6. Lagged (3-month lag) Spearman *rho* correlation between climate teleconnections (CT) and burned area (BA) during the fire season peak from 1982 to 2018.** The maps at 0.5º pixel resolution represent the correlations at P<0.05 (R = 0.34) and P<0.10 (R = 0.29) between BA and the studied CTs distributed throughout the globe (Arctic, Pacific, Atlantic, Indic and Antarctic) described in the methods section S.1.1. Atlantic Multidecadal Oscillation (AMO); Arctic Oscillation (AO); East Atlantic (EA); El Niño 3.4 SST index (ENSO); Eastern Pacific (EP); Indian Ocean Dipole (IOD); North Atlantic Oscillation (NAO); Pacific Decadal Oscillation (PDO); Pacific North American (PNA); Southern Annular Mode (SAM); Tropical North Atlantic (TNA); Tropical South Atlantic (TSA); Western Pacific (WP)


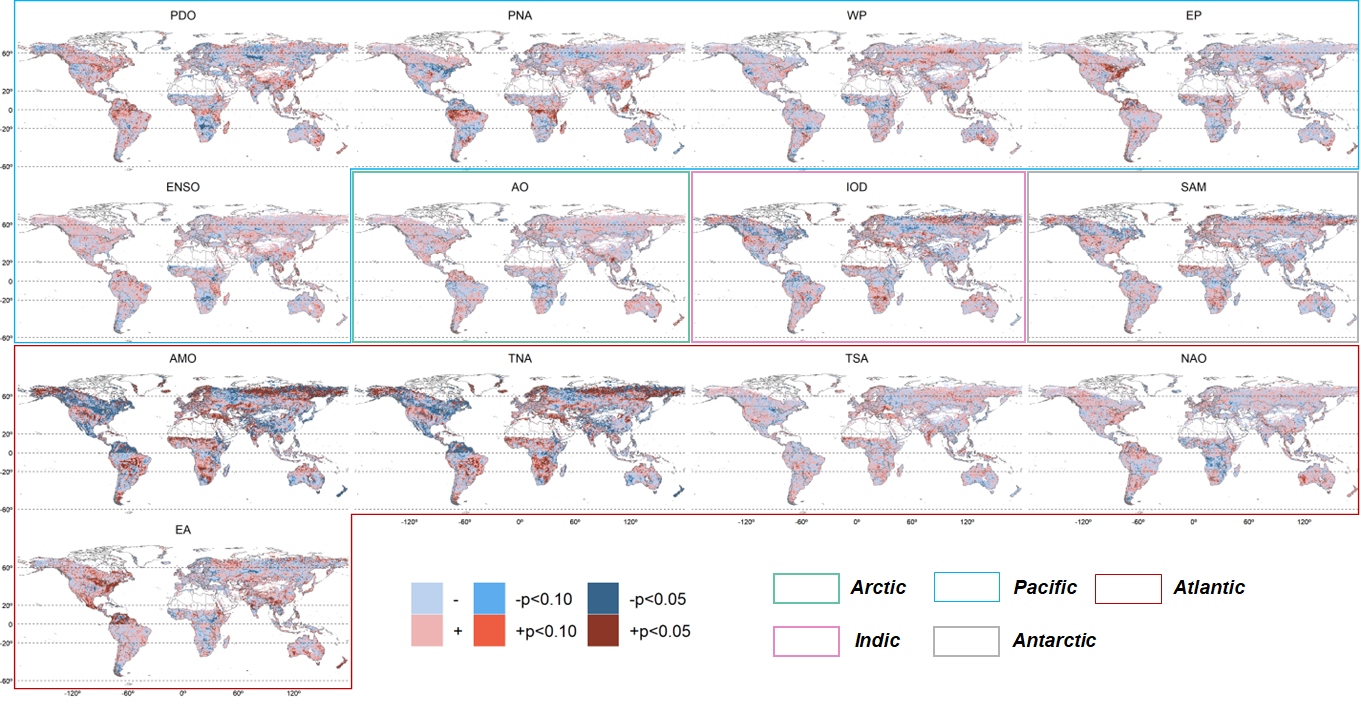


**Figure S7. Lagged (6-month lag) Spearman *rho* correlation between climate teleconnections (CT) and burned area (BA) during the fire season peak from 1982 to 2018.** The maps at 0.5º pixel resolution represent the correlations at P<0.05 (R = 0.34) and P<0.10 (R = 0.29) between BA and the studied CTs distributed throughout the globe (Arctic, Pacific, Atlantic, Indic and Antarctic) described in the methods section S.1.1. Atlantic Multidecadal Oscillation (AMO); Arctic Oscillation (AO); East Atlantic (EA); El Niño 3.4 SST index (ENSO); Eastern Pacific (EP); Indian Ocean Dipole (IOD); North Atlantic Oscillation (NAO); Pacific Decadal Oscillation (PDO); Pacific North American (PNA); Southern Annular Mode (SAM); Tropical North Atlantic (TNA); Tropical South Atlantic (TSA); Western Pacific (WP)


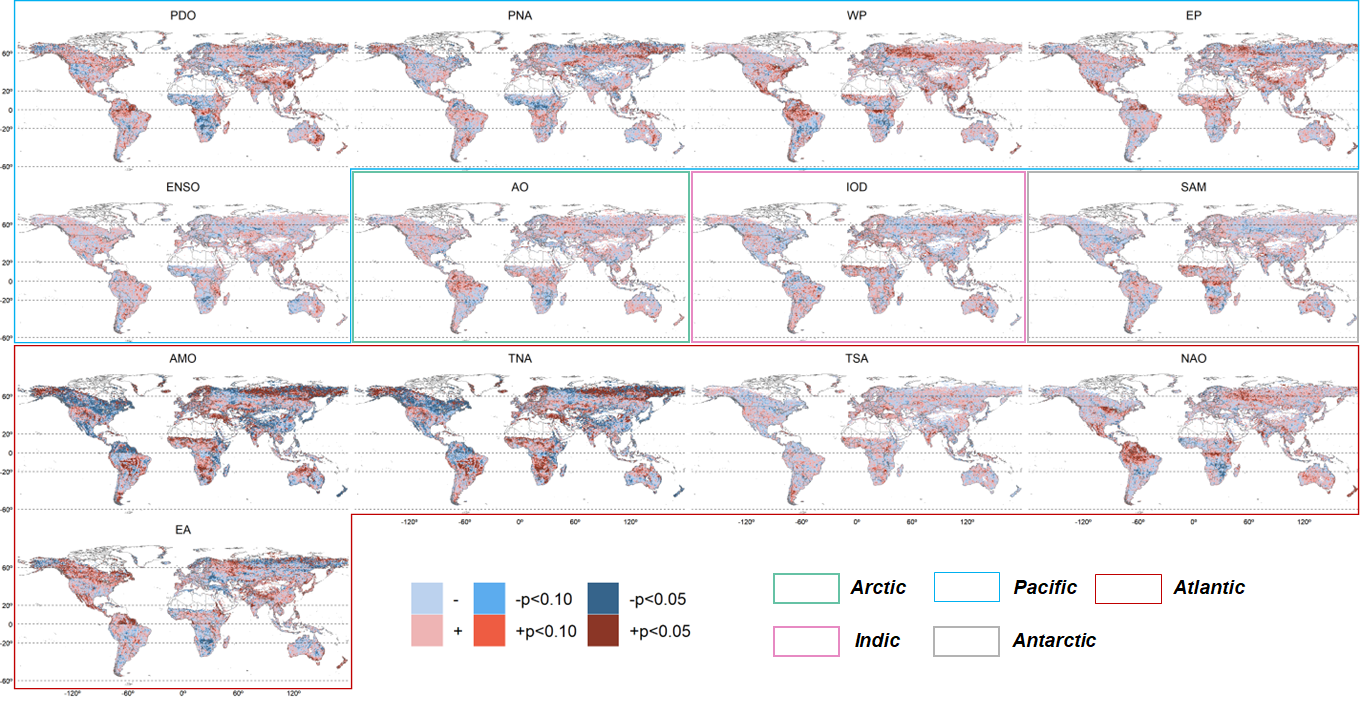


**Figure S8. Lagged (9-month lag) Spearman *rho* correlation between climate teleconnections (CT) and burned area (BA) during the fire season peak from 1983 to 2018.** The maps at 0.5º pixel resolution represent the correlations at P<0.05 (R = 0.34) and P<0.10 (R = 0.29) between BA and the studied CTs distributed throughout the globe (Arctic, Pacific, Atlantic, Indic and Antarctic) described in the methods section S.1.1. Atlantic Multidecadal Oscillation (AMO); Arctic Oscillation (AO); East Atlantic (EA); El Niño 3.4 SST index (ENSO); Eastern Pacific (EP); Indian Ocean Dipole (IOD); North Atlantic Oscillation (NAO); Pacific Decadal Oscillation (PDO); Pacific North American (PNA); Southern Annular Mode (SAM); Tropical North Atlantic (TNA); Tropical South Atlantic (TSA); Western Pacific (WP)

**Supplementary analysis on the representativeness of the fire season peak**

To disentangle the global relationships between climate teleconnections (CTs) and burned area (BA), we correlated both variables considering the fire season peak in the 1982-2018 period at 0.5º resolution, as described in the Methods section of this manuscript (S.1.1). In this section, we analyzed the temporal distribution of BA according to the fire season peak across the globe. A large percentage of the total annual BA (84.3 %) occurred during the peak as shown in Fig 1, indicating that our analysis thoroughly explains the linkages between BA and CT. The main global fire season peaks in terms of BA were related to boreal winter (NDJ and DJF) and boreal summer (JJA, JAS, ASO).

**
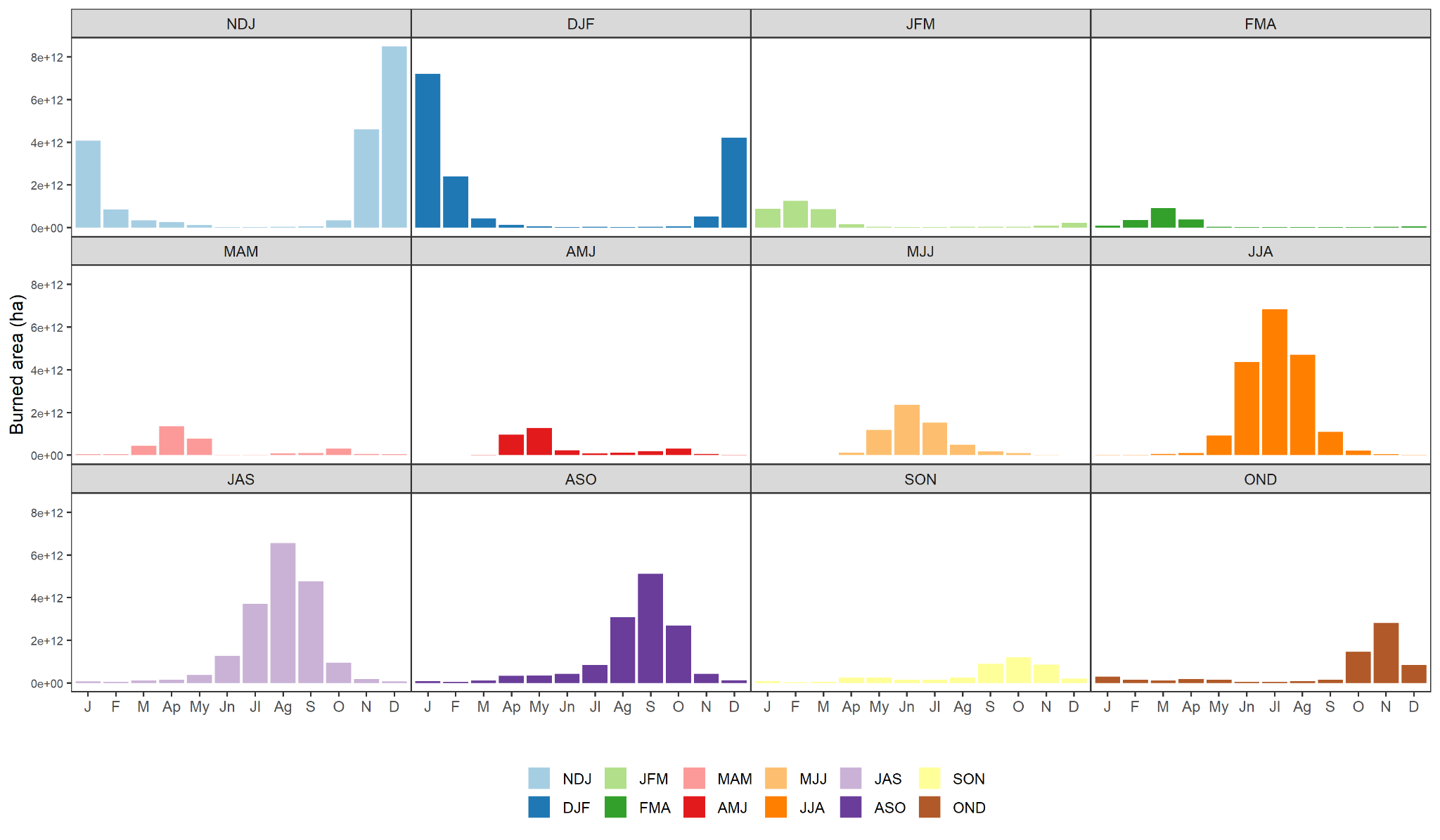
**

**Figure S9. Global average monthly burned area (BA) distribution across the different fire season peaks from 1982 to 2018.** During the peak of the fire season, fires burned 84.3 % of the total annual BA. The main global fire season peaks in terms of BA are related to winter (NDJ and DJF) and summer (JJA, JAS, ASO). The capital letters in both the legend and charts represent the initial letter of each month.

**Supplementary extended insights into global CT-fire relationships**

**Africa**

Africa accounts for about 70% of BA^1^ and 50% of global fire carbon emissions^2^. Correlations between weather patterns and some CTs have been recently investigated^3,4^ , but few studies have addressed the linkage between CTs and wildfires^5^. Most fires occurred in areas occupied by savannas within the 5 to 15°N latitude belt from November to February; and 10 to 20°S latitudes, with BA peaking from May to August (Fig 1). Savannas are characterized by a wet season of rapid fuel build-up (even in fire-affected areas) and a dry season capable of drying fuels out. Savanna fires in the northern African hemisphere (CTD 4) responded predominantly to +TNA (lag 0 and 3) and +AMO due to changes in atmospheric circulation driving lower precipitation^6^. The +SAM (lag 9) correlated with increasing BA likely due to the increased precipitation during the fuel build-up period[^7^](https://www.zotero.org/google-docs/?kbLkv8). EA conditioned both air temperature and rainfall in this region[^26^](https://www.zotero.org/google-docs/?BAvNGC) modulating BA (lag 9). +ENSO was found to promote below-average amounts of rainfall^4,8^, hence drier fuels and subsequently increased BA. Interestingly, this relationship was more evident at the 6-month lag, when the growing season of vegetation was just starting^9^. Likewise, the observed transitional area towards the Sahara Desert (Sahel; CTD 6), where BA decreased under +ENSO events, suggests that reduced rainfall could limit fuel availability in the fire season peak. Savanna fires in the southern African hemisphere (CTD 4) respond mainly to +AMO, +TNA (lag 0, 6 and 9) -SAM (lag 9), +PNA (lag 0), -PNA (lag and 3) and -NAO (lag 6 and 9). +SAM significantly reduced BA likely due to the increased precipitation and vegetation moisture^10^. +NAO (lag 6 and 9) was associated with reduced BA, which is consistent with the fact that it promotes wetter conditions prior to the fire season peak^3^. +PNA is associated with a reinforced jet stream from Asia potentially linked to above average convective precipitation over central Africa during the monsoon season thereby decreasing BA. This pattern is associated with the intensity of the Tropical Easterly Jet over Africa and the [Inter-tropical Convergence Zone (](https://www.eumetsat.int/inter-tropical-convergence-zone-itcz#:~:text=According%20to%20this%20scatterometer%20wind,in%20the%20RGB%20composite%20image.)ITCZ) position. Fire activity in eastern Africa (CTD 2) was mainly associated with +ENSO and +IOD. Both +IOD and +ENSO conditions relate to above-average rainfall in East Africa^8,11^, specifically in Kenya’s and Ethiopia’s grass-savannas from lag 0 to 6 months.

BA in tropical moist forests around the Equator peaks from July to September (Fig 1). Their shorter dry season and higher fuel moisture content hampers fire ignition and spread, resulting in much lower BA than in savannas. The tropical moist forests of the Congo Basin store substantial amounts of carbon while being one of the most biodiverse areas worldwide^12^. These forests are bordered by fire-prone savannas associated with deforestation from logging and land clearing^13^. The slightest increase in fire activity in this ecosystem may lead to large CO_2_ release and, hence, global impacts on climate dynamics as well as the loss of habitats^14^.Despite the attention this serious threat deserves, few publications have addressed this issue^15^. We found BA in this biome (CTD 3) to be mainly associated with -TNA (lag 0), PNA (lag 0, 3, and 9), +SAM (lag 9). Both +TNA and +PNA (in the fire season peak) promoted wetter conditions in the region, while the BA diminished accordingly. In contrast, +SAM promoted drought conditions some months before the fire season peak which may boost BA in these tropical moist forests^7^.

In southern Africa (CTD 1, 2 and 6; Figure 1D), we found significant correlations between BA and TNA, AMO, ENSO (lag 3, 6 and 9), PNA (lag 0), NAO (lag 9), AO (lag 9), EA (lag 0 and 9), EP (lag 0), IOD (lag 0 and 6), and SAM (lag 9), respectively. The +SAM promoted BA due to reduced rainfall in the austral summer in Angola (CTD 6), and PNA (lag 3) was positively correlated with BA because of increased precipitation before the fire season. NAO and AO influenced precipitation with a marked dipole in CTD 2 and 6. In CTD 6, precipitation during austral winter (lag 6 and 9) increased with +NAO, whereas the opposite occurs in CTD 2, influencing BA accordingly. +ENSO, likely coupled with +PDO, decreased BA in the CTD 6 due to a negative correlation with rainfall during March and July^3^.

**Americas**

Wildfires in the Americas affect a diverse range of biomes - from boreal forests of Canada to temperate forests of the southeastern United States and tropical rainforests in the Amazon basin - each displaying unique fire regimes and seasonal timing. Most of the fires within this region occur in the tropical savanna, shrubland and grasslands to the north (Colombian Llanos, CTD 3) and southeast of the Amazon (Cerrado, CTD 6). The fire season peak in the tropical savanna spans from February to April in the northern Llanos and from August to October in the southern Cerrado (Fig. 1). BA follows a seasonal pattern matching the growth cycle of herbaceous communities during the wet season that rapidly dries out during the dry season, leading to fire-prone conditions^16^. Teleconnections such as +PDO and +ENSO concur in correlation with increased BA within this biome, linked to anomalously warm and dry conditions^10^. The synchronous +EA pattern (lag 0) decreases BA in the southern Amazonian Cerrado^17^. The IOD+ and the SAM+ are both associated with reduced BA by fostering precipitation^18^. +TNA and +AMO drive increases in BA in CTD 6 due to significant increases in terrestrial evaporation during and before the fire season peak^11^

The Amazon Rainforest (CTD 2) is revered as the unrivaled biodiversity hotspot featuring the largest collection of terrestrial plant and animal species found anywhere in the world^19^. Fires in this vulnerable biome are rare with return intervals of hundreds -if not thousands- of years and are usually prompted by deforestation and moisture stress as a result of extensive drought^14,20^. We found synchronous (PNA, EP, TNA and AMO) and lagged signals associated with extreme drought conditions and fuel aridity in the Amazon including the AO, EA, NAO, SAM and the NAO at 9-month lags^18^. The Atlantic influence is through changes in the north-south divergent circulation and the movement of the ITCZ following warm SST^21^ . Therefore, association is stronger in the southern part of the Amazon basin during the fire season peak^22^. Droughts and BA in this region are mediated by +TNA and +AMO associated with a sustained northward position of the ITCZ^21^. On the other hand, +PNA is accompanied by positive precipitation anomalies -diminishing fire activity- across the Amazon in the austral summer (lag 0)^23^. Unexpectedly, our results indicate that the correlation between ENSO and BA was not significant within the Amazon despite documented precipitation deficits during +ENSO^18^. This could be related to underlying factors leading to increased BA^24^ and higher rates of evapotranspiration before the fire season peak during -ENSO^11^.

The southern end of Patagonia and the Pampas is characterized by a temperate climate, hosting grasslands and broadleaved and mixed forests (CTD 2 and 6). Compared to other biomes, the percentage of annual BA occurring during the fire season peak was lower, probably explaining the weaker correlation between CTs and BA. The +SAM is known to boost wildfire activity in the mid latitude ranges in South America by promoting significantly warmer and dryer conditions during the austral summer, especially in the Chilean Patagonia^10,25^. Our study captures some of the respective increases in BA in this region during the 0-month lag. However, most of the notable increases in BA are identified further east in the Pampas region of Argentina and Uruguay (CTD 6), likely linked to intense drought conditions^18^. While ENSO also has been shown to be positively correlated with BA in southwest South America as well as in the mediterranean and temperate regions of Chile, our results only found significant the ENSO-BA correlation at 9-month lags in Uruguay, north of Argentina and south of Brazil^5,25^ Both TNA and AMO also modulate BA through a north-south dipole in Argentina^26^.

Wildfires within the North American taiga are a dominant disturbance shaping landscape diversity across the boreal forests of Alaska and Canada^27,28^. The influence of CTs on wildfire activity have been thoroughly studied in this region, with the peak of the fire season occurring from June to September. Several studies identified PDO and ENSO as the main CTs driving fire activity in this region^24,29–32^. +PDO and +ENSO are associated with anomalously warmer and drier conditions across all of the Pacific Northwest and Canada, particularly from November to April ^18,33–36^. These more arid conditions are likely responsible for the observed increase in BA at the 9-month lag. IOD+ also appears to be a driving factor of BA increase in the taiga. Saji & Yamagata (2003)^37^ investigated the impacts of IOD on the global climate, and identified above-average surface temperature anomalies across northern North America during the summer months and beginning of autumn, which appear to be responsible for the widespread increase in BA fostered by the 9-month lag IOD pattern. +SAM is known to drive cooler temperatures across northern latitudes in North America, which is reflected in the decrease in BA at the 9-month lag^23^. Both Atlantic (EA, AO and NAO) and Pacific (EP and WP) CT modes correlate with BA at different time lags based on synchronous and lagged relationships between terrestrial evapotranspiration and CTs found by previous research^11^.

Wildfires in California and the Pacific Southwest have long been considered endemic to the region. This is partly due to the confluence of dry, windy and hot weather conditions with accumulation of fuels, growing population, and infrastructures, which have resulted in a steadily high frequency and intensity of wildfires over the years^38^. Our results struggled to identify the influential CTs identified in other studies. This may be due in part to the relatively small size of the region. There have been several studies positioning the PDO as a key regulator of fire regimes in the Pacific Northwest through its influence on fuel production and succession dynamics^39–41^. Within North America, +ENSO is known to induce contrasting precipitation anomalies promoting warmer and drier conditions during the cool season in the northwest, and wetter in the southwest^42,43^. Barbero et al. (2015)^44^ linked increases in summertime precipitation associated with +ENSO with decreases in BA in the nearby region of the Great Basin and Northern Rockies; a relationship that has strengthened in recent decades. While Cardil et al. (2021)^45^ identified striking connections between drought and ENSO in southern California during autumn, we identified no significant linkages between BA and ENSO in the fire season peak, probably due to the fact that ENSO is weaker during the summer season compared to the autumn. Despite the lack of significant correlations between BA and ENSO along the Pacific Coast, some previous research discloses that, when synchronized with +PDO, +ENSO tends to boost BA, particularly in northern California, Oregon and Washington States^39,45,46^, whereas Barbero et al., (2015)^44^ found decreases in BA when +ENSO persists into the summer. Our results indicate that +AMO and +TNA both boost BA across the Pacific Northwest coast of the US.

The southeastern United States hosts a number of primarily temperate ecoregions including the coastal plains and the southern Appalachian mountains. Most ecosystems exhibit low intensity surface fires^47^. The positive phases of NAO, EA and ENSO all resulted in positive correlations with BA. The positive phases of AMO and TNA have all been reported to produce cooler and wetter conditions across southeastern United States, particularly in the late winter and early spring, owing to the shift of storm tracks in the Atlantic influencing moisture availability^48–50^. These patterns are in agreement with our results that identified decreases in BA during +TNA and +AMO. The pacific CT modes (+EP and +WP) are associated with increased BA through lagged effects (6 and 9 months).

Southern Mexico and Central America (CTD 5) are composed of tropical and subtropical rainforests, much of which are susceptible to fire predominantly burning in late spring^51^ (Fig. 1). +PDO, and +ENSO further threaten the ecosystems in this region inducing increases in BA according to our statistical analyses. During +ENSO events, the region is usually dry; the mid-summer drought is strong and likely responsible for the heightened BA^52^. Maldonado et al. (2016)^53^ determined that +ENSO are associated with drier mid-summer drought across Central America. Our results also highlight decreases in BA during positive phases of EA (lag 6 and 9), EP (lag9), WP (lag 9) and NAO (lag 9). +AMO and +TNA are associated with decreased BA probably due to increased precipitation in the region^54^.

**Asia**

Asia has several major burned area hotspots mainly located in the temperate steppe (central Asia), the tropical and subtropical dry forests of southeastern Asia (India, Thailand) and the temperate broadleaf forests of China. The CTDs we identified clearly represent the most distinctive CTs driving wildfire activity at the continental scale. BA in southern Asia (CTD 5 and 6) is largely modulated by -ENSO (lags 0, 3 and 6), -PDO, -IOD (lag 3), AMO, TNA, +SAM (lags 0 and 3), TSA (lag 6 and 9), +EA (lag 6), -EP (lag 3) and +WP (lag 6 and 9), with a fire season extending from January to May varying latitudinally across the continent(Fig 1). It is known that the simultaneous occurrence of +ENSO events from June to September reduces the duration of the Indian summer monsoon rainfall (ISMR) and, to some extent, the onset period^55^. In turn, preceding winter -ENSO also shortens the ISMR, especially in western and southern India, and promotes wetter conditions during the fire season peak^56,57^. This behavior matches the negative relationship between ENSO and wildfire activity at lags 0, 3 and 6. The IOD also affects the ISMR on its own and modulates the influence of the ENSO on the ISMR^58^. Generally, +IOD facilitates extraordinary rainfalls over India during the ISMR, decreasing burned areas in the fire season peak^58^. AMO and TNA also modulated BA through a dipole between CTD 6 and 5 in India due to their influence on the ISMR due to shifts in the ITCZ and tropical interbasin interactions with the Pacific Ocean^59^. +AMO could induce increased summer rainfall in central and southern India^60^.

Southeastern Asia is composed of two different areas: [Maritime and Mainland SE Asia](https://en.wikipedia.org/wiki/Mainland_Southeast_Asia), with relatively higher fire frequencies found for Myanmar, Thailand, Cambodia, and Laos^61^. Rates of deforestation and forest degradation are among the highest in this part of the world, and fires play a considerable role due to slash and burn practices for agricultural expansion and timber extraction^62,63^. BA across mainland SE Asia are driven by +ENSO (lag 0, 3) +PDO, +IOD (lag 3, 6 and 9), +EP (lag 9), +TNA (lag 0, 3), +AMO and +EA (lag 3). +PDO influences the East Asian Summer Monsoon and produces wetter conditions in Myanmar and drier conditions across the southeastern regions of the Indochinese Peninsula^64^, which is reflected in the differences in BA observed in our analyses. +IOD restricts rainfall in June-August across mainland SE Asia, particularly in Cambodia and central Laos, where we witness the greatest BA increases^64,65^ . Similarly, +ENSO amplifies frequency, duration and intensity of heat waves^66^ and drought in much of Indochina, therefore driving increases in BA^67,68^. +AMO and +TNA promote increased BA across mainland SE Asia.

Wildfire activity in the [Maritime](https://en.wikipedia.org/wiki/Mainland_Southeast_Asia) region is a result of the interplay of land use practices and climate^69^ with a fire season peak from June to September. A substantial percentage of BA relates to peatland fires in southern Sumatra and southern Kalimantan. These fires are of major concern for their adverse impacts on air quality, as emanating toxic haze induces respiratory problems and even death among vulnerable populations leaving Indonesia as the region with the highest concentration of fire-related pollution^70,71^. BA was predominantly associated with drought mediated by the positive phases of ENSO (lag 0, 3 and 6), TNA (lag 0), IOD (lag 0), PNA (lag 6), WP (lag 9)^72,73^. When both +IOD and +ENSO occur simultaneously, droughts during the dry season are amplified and wildfire activity is heightened^72–77^, particularly in areas dominated by savannas and croplands such as the islands of Java and southern Borneo. Interestingly, opposing trends were observed in the tropical evergreen broadleaf forest, especially across the islands of New Guinea and the northern region of Borneo, where BA was suppressed at the 0- and 3-month lags.

Eastern Asia (China) holds the world’s largest subtropical forests, where fires are modulated by the Asian summer monsoon in mountainous areas. The region falls within the CTDs 1, 2, 3 and 6. Most of the BA occurred in subtropical China (~20–30°N, 100–120°E) during the dry season^78^. The fire season peak in eastern Asia varies latitudinally. In the south, the fire season starts earlier (from January to March), while BA in the northernmost region of China, at the limits of the summer monsoon, peaks during summer (June to August). In southern China, Fang et al. (2021)[^78^](https://www.zotero.org/google-docs/?tRtyv7) found a fire occurrence dipole between southwestern and southeastern China mediated by ENSO. Although we did not observe this dipole in BA in agreement with recent research^79^, we found significant effects of +ENSO on BA (lag 6 and 9), probably amplified by +PDO^80^. Reduction in BA was found under +AMO and +TNA (lag 0) due to enhanced precipitation over the region^81^. Also, the +EA (lag 0), +EP (lag 0) and +WP (lag 9) are associated with increased BA, all these driving weather conditions of limited precipitation, high temperature and dry fuels during fire seasons^82,83^.

The Asian Boreal taiga is a vast landscape dominated by coniferous forests extending across Eurasia, being a classic example of a fire-dependent ecosystem. Fires are an important natural disturbance in the taiga, responsible for forest succession and regeneration. Based on our results, the taiga biome can be classified into two dominant CTDs: CTD 6 in the west across the Ural Mountains, and CTD 5 in eastern Siberia. Siberia (CTD 5) appears to be susceptible to increases in BA during +NAO, +AO, +PNA (lag 3, 6) and +IOD (lag 0) events. We also observed similar impacts of AO and NAO (both highly correlated, partly due to shared storm tracks) between the northeastern Atlantic and Arctic regions during the wintertime^84^. However, an anticipated warmer climate is expected for AO to shift further from NAO^85^. AO is known to be associated with climate variability across Eurasia, with significant influence on temperature. Indeed, it has been found to drive extremely high temperatures in late winter causing premature snowmelt, eventually boosting wildfire activity^86–88^. NAO was found to be positively correlated with BA at 9-month time lag, which may indicate fuel build-up connecting to fire activity within the Siberian taiga. The winter +NAO is associated with heightened springtime vegetation growth activity throughout Eurasia, which we believe explains the observed phase transition^89^. Studies have also suggested that the influence of ENSO on precipitation may drive the wildfire regime in eastern Siberia as reflected by our statistical analysis at 6- and 9-month lags^88^. Reductions in BA are correlated with the positive phases of EA (lag 0, 3), EP (lag 0), WP (lag 0) due to decreased evapotranspiration during the fire season peak from April to June^11^. The western taiga (CTD 6) experiences heightened BA during the positive phase of AO (lag 0 ,3 and 9), NAO (lag 3 and 9), EA (lag 9), WP (lag 6 and 9) and SAM (lag 6). Reductions in BA are correlated with the positive phases of IOD (lag 0, 3 and 6) and AMO. Both NAO and EA are closely tied to precipitation anomalies across this region during the fire season peak^11,90^ and gradually become more dominant in summertime [as reflected in our results (lag 9)](https://www.zotero.org/google-docs/?CkDgIT). In the northeast of Russia, terrestrial evaporation is sensitive to the IOD, and WP patterns, inducing variability in air temperatures across the region^11^.

The Eurasian Steppe (or Great Steppe) is among the largest grasslands in the world, extending from Hungary to China. This region has become a hotspot of wildfire activity in the past few decades following a rural exodus after the dissolution of the Soviet Union^91,92^. Previous research indicates a sevenfold increase in BA between 1990-2000, an intensified fire regime shift associated with higher fuel loads from limited grazing pressures^91^. Around 90% of BA in Central Asia is located within Kazakhstan^93^. Fires within these temperate grasslands peak from August to October in the CTD 2 and April to June in the CTD 6 (northern Kazakhstan). AMO, TNA, PNA and EA are the most prominent teleconnections sustaining wildfire activity in this region in agreement with previous research^17^. Spring EA is associated with decreased summertime precipitation and reduced aboveground net primary productivity across northern Mongolia Plateau grasslands^94^. After a previous warm winter AMO, it has been observed a significantly higher sea level pressure and geopotential height in the summer of Eurasia associated with descending atmospheric motion, which could lead to adiabatic warming. This mechanism leads to increased incoming solar energy and warming rate, enhancing the risk of wildfires^17^.

**Europe**

Approximately, half a million hectares of forest and wildlands are burned across Europe every year. More than 85% of the burned area is located in southern Europe, a Mediterranean climate-type area prone to fire^95^. The Mediterranean region is characterized by a strong seasonal gradient with cool-and-wet winters fostering vegetation growth, and hot dry summers heightening vegetation flammability. The fire season typically peaks from July to September (Fig 1). Mediterranean fires respond predominantly to AMO, TNA, TSA, EA, PDO, IOD, and ENSO. Previous studies linking IOD and ENSO teleconnections to weather conditions in the Mediterranean report anomalies in temperature and precipitation which support fuel build up in the wet season which later burns in the dry season, which is reflected in our results^96,97^. AMO and TNA (lag 3, 6 and 9) were predominantly associated with increased BA in Greece and Turkey. EA is associated with higher temperatures throughout Europe all year long and decreased precipitation in the south which is reflected in the positive association between the index and BA in Spain and France.

Temperate continental Europe is relatively more densely populated than the rest of the continent; marked by a long-standing human presence and urban sprawl that has resulted in a fragmented and mosaic land. As a result, BA in this region is generally sparser, posing a challenge when investigating trends. Similarly, to the Mediterranean area, this temperate region responds to +AMO, -NAO (lag 3) +PDO, +ENSO (lag 6 and 9), +EA (lag 9) and +IOD (lag 6), all driving increases in BA. +NAO suppresses BA in this region particularly at the 3-month lag owing to increased westerly winds, which bring moist air into Europe producing mild winters accompanied with frequent rain^98,99^. EA is also among the most discussed CTs within Europe, often in its connection with NAO, and their combined influence on the variability of the North Atlantic eddy-driven jet stream^100^. On its own, EA is associated with higher temperatures throughout Europe all year long, increased precipitation in northwest Europe. Our statistical analysis highlights increases in BA across central and western Europe at the 9-month lag, likely due to warm temperature and increased evapotranspiration before the fire season peak^101^.

The Scandinavian boreal landscape is dominated by coniferous forests and tundra; both biomes are strongly tied to the carbon cycle for their abundant peatlands and their key role in carbon sequestration, an ecosystem function under threat of increased warming^102,103^. Forest fires are among the main disturbances in this region and have been long linked to summer drought conditions, showing strong correlations between annually burned forest areas and drought indices^104^. As mentioned above, NAO is associated with above normal precipitation over northern Europe and Scandinavia due to the change in the Atlantic jet stream. The summer NAO is known to be associated with European blocking events which may produce heatwaves and drought conditions, and have been linked to increased fire activity within the European Boreal Zone^105^. Our statistical results reveal decreased BA during the positive phase of NAO (lag 3) in southern Scandinavia.

**Oceania**

The recent extraordinary Australian wildfires showed how climatic events can cause unprecedented large-scale impacts through the combination of sustained record-breaking high temperatures with low precipitation^106,107^. Two main CTDs are associated with the spatial relationships between CTs and BA across this continent: CTD 2 in central and southern Australia and CTD 6 in northern Australia, where most of the BA occurred in the tropical savanna, shrubland and grasslands (Fig. 1). In this continent, the equatorial Pacific SST variability is the main driver of observed precipitation variability although the Indic and Atlantic Ocean also impact on Australian precipitation and temperature variability by affecting changes in the tropical Pacific climate through the trans-basin variability mechanism^108,109^. Precipitation is enhanced over northern Australia by +PDO in contrast to the eastern and western Australia where it is weakened^110^. +IOD brings easterly wind anomalies across the Indian Ocean, less cloudiness to Australia's northwest, less rainfall and increased evapotranspiration over southern and northern Australia^111^. The lags between the IOD and BA are consistent with the effects of this CT index on weather conditions before the fire season peak. Both TSA, TNA and AMO drive changes in temperature and precipitation patterns across Australia^11,108,109,112^. Previous research identified a decadal-scale inter-hemispheric SST dipole mode in the Atlantic ocean, which is mainly characterized by SST anomalies of opposing sign between the North and South the Ocean^109^ and how these SST patterns may affect weather conditions in Australia through a prominent eastward propagation originating from the South Atlantic, which is demonstrated to be Rossby wave response to the South Atlantic multidecadal variability forcing. All these CT-weather patterns are reflected in the associations between BA and CTs in our study in agreement with previous research^17^. The TSA positively modulated BA in SE Australia probably due to increased surface air temperature during the Austral summer. As expected, the effects of TNA and AMO compared to TSA in this region had the opposite sign.

In summary, in northern Australia (CTD 6), BA is mainly modulated by +AMO, +TNA (lag and 9), +IOD (lag 0, 3 and 6) and -PDO. In the CTD 2, in addition to the effects of the aforementioned CTs, we found more CT-fire patterns including the effect of +ENSO in SE Australia that brings increased surface air temperature and drought conditions^106,113^, an effect potentially coupled with +IOD^111^. Also, the Pacific (+EP lag 3, +WP lag 6 and PNA lag 3 and 6) and Atlantic (+EA lag 3 and 6, NAO lag 6) CTs appear to modulate increases in BA in the CTD 4 as found by^17^. +SAM (lag 6) was found to be negatively correlated with BA due to coinciding cool and wet conditions over SE Australia^114^.

**Supplementary References**

1. Giglio, L., Boschetti, L., Roy, D. P., Humber, M. L. & Justice, C. O. The Collection 6 MODIS burned area mapping algorithm and product. *Remote Sensing of Environment* **217**, 72–85 (2018).

2. Van Der Werf, G. R. *et al.* Global fire emissions estimates during 1997-2016. *Earth System Science Data* **9**, 697–720 (2017).

3. Lüdecke, H.-J., Müller-Plath, G., Wallace, M. G. & Lüning, S. Decadal and multidecadal natural variability of African rainfall. *Journal of Hydrology: Regional Studies* **34**, 100795 (2021).

4. Nash, D. J. & Adamson, G. C. Recent advances in the historical climatology of the tropics and subtropics. *Bulletin of the American Meteorological Society* **95**, 131–146 (2014).

5. Mariani, M., Veblen, T. T. & Williamson, G. J. Climate Change Amplifications of Climate-Fire Teleconnections in the Southern Climate Change Ampli fi cations of Climate-Fire Teleconnections in the Southern Hemisphere. *Geophysical Research Letters* **45**, (2018).

6. Nouaceur, Z. & Murarescu, O. Rainfall variability and trend analysis of rainfall in West Africa (Senegal, Mauritania, Burkina Faso). *Water* **12**, 1754 (2020).

7. Purich, A., Cowan, T., Min, S.-K. & Cai, W. Autumn Precipitation Trends over Southern Hemisphere Midlatitudes as Simulated by CMIP5 Models. *Journal of Climate* **26**, 8341–8356 (2013).

8. Moore, S. M. *et al.* El Niño and the shifting geography of cholera in Africa. *Proceedings of the National Academy of Sciences* **114**, 4436–4441 (2017).

9. Winkler, K., Gessner, U. & Hochschild, V. Identifying droughts affecting agriculture in Africa based on remote sensing time series between 2000–2016: rainfall anomalies and vegetation condition in the context of ENSO. *Remote Sensing* **9**, 831 (2017).

10. Gillett, N. P., Kell, T. D. & Jones, P. D. Regional climate impacts of the Southern Annular Mode. *Geophysical Research Letters* **33**, (2006).

11. Martens, B., Waegeman, W., Dorigo, W. A., Verhoest, N. E. & Miralles, D. G. Terrestrial evaporation response to modes of climate variability. *NPJ Climate and Atmospheric Science* **1**, 1–7 (2018).

12. Lewis, J. & Nelson, J. Logging in the Congo Basin. What hope for indigenous peoples’ resources, and their environments? *Indigenous affairs* **4**, 8–15 (2006).

13. Bucini, G. & Lambin, E. F. Fire impacts on vegetation in Central Africa: a remote-sensing-based statistical analysis. *Applied Geography* **22**, 27–48 (2002).

14. Cardil, A. *et al.* Recent deforestation drove the spike in Amazonian fires. *Environmental Research Letters* **15**, 121003 (2020).

15. Juarez, S., Siebe, C. & Fernández y Fernández, D. Causes and Effects of Forest Fires in Tropical Rainforests: A Bibliometric Approach. *Tropical Conservation Science* **10**, 194008291773720 (2017).

16. Romero‐Ruiz, M., Etter, A., Sarmiento, A. & Tansey, K. Spatial and temporal variability of fires in relation to ecosystems, land tenure and rainfall in savannas of northern South America. *Global Change Biology* **16**, 2013–2023 (2010).

17. Shi, K. & Touge, Y. Characterization of global wildfire burned area spatiotemporal patterns and underlying climatic causes. *Scientific Reports* **12**, 644 (2022).

18. Reboita, M. S. *et al.* Impacts of teleconnection patterns on South America climate. *Annals of the New York Academy of Sciences* **1504**, 116–153 (2021).

19. Myers, N. *The primary source: tropical forests & our future.* (WW Norton & Company, Inc., 1992).

20. Kauffman, J. B. & Uhl, C. Interactions of anthropogenic activities, fire, and rain forests in the Amazon Basin. in *Fire in the tropical biota* 117–134 (Springer, 1990).

21. Towner, J. *et al.* Influence of ENSO and tropical Atlantic climate variability on flood characteristics in the Amazon basin. *Hydrology and Earth System Sciences* **25**, 3875–3895 (2021).

22. Wang, H. & Fu, R. The Influence of Amazon Rainfall on the Atlantic ITCZ through Convectively Coupled Kelvin Waves. *Journal of Climate* **20**, 1188–1201 (2007).

23. Kistler, R. *et al.* The NCEP-NCAR 50-Year Reanalysis: Monthly Means CD-ROM and Documentation. *Bulletin of the American Meteorological Society* **82**, 247–267 (2001).

24. Macias Fauria, M. & Johnson, E. A. Large‐scale climatic patterns control large lightning fire occurrence in Canada and Alaska forest regions. *Journal of Geophysical Research: Biogeosciences* **111**, (2006).

25. Holz, A. *et al.* Southern Annular Mode drives multicentury wildfire activity in southern South America. *Proceedings of the National Academy of Sciences* **114**, 9552–9557 (2017).

26. Pérez, S., Sierra, E., Momo, F. & Massobrio, M. Changes in average annual precipitation in Argentina’s Pampa region and their possible causes. *Climate* **3**, 150–167 (2015).

27. Erni, S., Arseneault, D., Parisien, M.-A. & Bégin, Y. Spatial and temporal dimensions of fire activity in the fire‐prone eastern Canadian taiga. *Global Change Biology* **23**, 1152–1166 (2017).

28. Stocks, B. J. *et al.* Large forest fires in Canada, 1959–1997. *Journal of Geophysical Research* **108**, 1–12 (2003).

29. Kitzberger, T., Brown, P. M., Heyerdahl, E. K., Swetnam, T. W. & Veblen, T. T. Contingent Pacific-Atlantic Ocean influence on multicentury wildfire synchrony over western North America. *Proceedings of the National Academy of Sciences* **104**, 543–548 (2007).

30. Goff, H. L. *et al.* Historical fire regime shifts related to climate teleconnections in the Waswanipi area, central Quebec, Canada. *International Journal of Wildland Fire* **16**, 607–618 (2007).

31. Skinner, W. R., Shabbar, A., Flannigan, M. D. & Logan, K. Large forest fires in Canada and the relationship to global sea surface temperatures. *Journal of Geophysical Research: Atmospheres* **111**, (2006).

32. Meyn, A., Taylor, S. W., Flannigan, M. D., Thonicke, K. & Cramer, W. Relationship between fire, climate oscillations, and drought in British Columbia, Canada, 1920–2000. *Global Change Biology* **16**, 977–989 (2010).

33. Newman, M. *et al.* The Pacific decadal oscillation, revisited. *Journal of Climate* **29**, 4399–4427 (2016).

34. Whitfield, P. H., Moore, R. D., Fleming, S. W. & Zawadzki, A. Pacific decadal oscillation and the hydroclimatology of western Canada—Review and prospects. *Canadian Water Resources Journal* **35**, 1–28 (2010).

35. Ropelewski, C. F. & Halpert, M. S. North American Precipitation and Temperature Patterns Associated with the El Niño/Southern Oscillation (ENSO). *Monthly Weather Review* **114**, 2352–2362 (1986).

36. Dai, A. & Wigley, T. M. L. Global patterns of ENSO-induced precipitation. *Geophysical Research Letters* **27**, 1283–1286 (2000).

37. Saji, N. & Yamagata, T. Possible impacts of Indian Ocean Dipole mode events on global climate. *Climate Research* **25**, 151–169 (2003).

38. Keeley, J. E. & Syphard, A. D. Twenty-first century California, USA, wildfires: fuel-dominated vs. wind-dominated fires. *Fire Ecology* **15**, 1–15 (2019).

39. Norman, S. P. & Taylor, A. H. Tropical and north Pacific teleconnections influence fire regimes in pine-dominated forests of north-eastern California, USA. *Journal of Biogeography* **30**, 1081–1092 (2003).

40. Hessl, A. E., McKenzie, D. & Schellhaas, R. Drought and Pacific Decadal Oscillation linked to fire occurrence in the inland Pacific Northwest. *Ecological applications* **14**, 425–442 (2004).

41. Gedalof, Z., Peterson, D. L. & Mantua, N. J. Atmospheric, climatic, and ecological controls on extreme wildfire years in the northwestern United States. *Ecological Applications* **15**, 154–174 (2005).

42. Dettinger, M. D., Cayan, D. R., Diaz, H. F. & Meko, D. M. North–south precipitation patterns in western North America on interannual-to-decadal timescales. *Journal of Climate* **11**, 3095–3111 (1998).

43. Gershunov, A. & Barnett, T. P. Interdecadal modulation of ENSO teleconnections. *Bulletin of the American Meteorological Society* **79**, 2715–2726 (1998).

44. Barbero, R., Abatzoglou, J. T. & Brown, T. J. Seasonal reversal of the influence of El Niño–Southern Oscillation on very large wildfire occurrence in the interior northwestern United States. *Geophysical Research Letters* **42**, 3538–3545 (2015).

45. Cardil, A. *et al.* Coupled effects of climate teleconnections on drought, Santa Ana winds and wildfires in southern California. *Science of the Total Environment* **765**, (2021).

46. Westerling, A. L. & Swetnam, T. W. Interannual to decadal drought and wildfire in the western United States. *EOS, Transactions American Geophysical Union* **84**, 545–555 (2003).

47. Mitchell, R. J. *et al.* Future climate and fire interactions in the southeastern region of the United States. *Forest Ecology and Management* **327**, 316–326 (2014).

48. Liu, Z. *et al.* Recent contrasting winter temperature changes over North America linked to enhanced positive Pacific-North American pattern. *Geophysical Research Letters* **42**, 7750–7757 (2015).

49. Henderson, K. G. & Robinson, P. J. Relationships between the pacific/north american teleconnection patterns and precipitation events in the south-eastern USA. *International Journal of Climatology* **14**, 307–323 (1994).

50. Brenner, J. Southern Oscillation Anomalies and Their Relationship to Wildfire Activity in Florida. *Int. J. Wildland Fire* **1**, 73–78 (1991).

51. Rodríguez Trejo, D. A. Fire regimes, fire ecology, and fire management in Mexico. *Ambio* **37**, 548–556 (2008).

52. Curtis, S. Interannual variability of the bimodal distribution of summertime rainfall over Central America and tropical storm activity in the far-eastern Pacific. *Climate Research* **22**, 141–146 (2002).

53. Maldonado, T., Rutgersson, A., Alfaro, E., Amador, J. & Claremar, B. Interannual variability of the midsummer drought in Central America and the connection with sea surface temperatures. *Advances in Geosciences* **42**, 35–50 (2016).

54. Montero-Martínez, M. J., Pita-Díaz, O. & Andrade-Velázquez, M. Potential Influence of the Atlantic Multidecadal Oscillation in the Recent Climate of a Small Basin in Central Mexico. *Atmosphere* **13**, (2022).

55. Moron, V. & Robertson, A. W. Interannual variability of Indian summer monsoon rainfall onset date at local scale. *International journal of climatology* **34**, 1050–1061 (2014).

56. Chakraborty, A. Preceding winter La Niña reduces Indian summer monsoon rainfall. *Environmental Research Letters* **13**, 054030 (2018).

57. Yadav, R. K., Ramu, D. A. & Dimri, A. P. On the relationship between ENSO patterns and winter precipitation over North and Central India. *Global and planetary change* **107**, 50–58 (2013).

58. Ashok, K., Guan, Z. & Yamagata, T. Impact of the Indian Ocean dipole on the relationship between the Indian monsoon rainfall and ENSO. *Geophysical research letters* **28**, 4499–4502 (2001).

59. Svendsen, L. The Atlantic Multidecadal Oscillation and Indian summer monsoon variability: a revisit. in *Indian Summer Monsoon Variability* 353–374 (Elsevier, 2021).

60. Shi, W., Wang, Q., Xiao, Z., Cheng, W. & Duan, W. Modulation of Atlantic Multidecadal Oscillation on the Interdecadal Variation of South Asian High and Somali Jet in Summer. *Changes in Snow, Monsoon and Snow-Monsoon Relationship in the Warming Climate* (2022).

61. Vadrevu, K. P. *et al.* Trends in vegetation fires in south and southeast Asian countries. *Scientific reports* **9**, 1–13 (2019).

62. Geist, H. J. & Lambin, E. F. Proximate Causes and Underlying Driving Forces of Tropical DeforestationTropical forests are disappearing as the result of many pressures, both local and regional, acting in various combinations in different geographical locations. *BioScience* **52**, 143–150 (2002).

63. Langner, A., Miettinen, J. & Siegert, F. Land cover change 2002–2005 in Borneo and the role of fire derived from MODIS imagery. *Global Change Biology* **13**, 2329–2340 (2007).

64. Hong, C.-C., Lu, M.-M. & Kanamitsu, M. Temporal and spatial characteristics of positive and negative Indian Ocean dipole with and without ENSO. *Journal of Geophysical Research: Atmospheres* **113**, (2008).

65. Gao, Q.-G., Sombutmounvong, V., Xiong, L., Lee, J.-H. & Kim, J.-S. Analysis of drought-sensitive areas and evolution patterns through statistical simulations of the Indian Ocean Dipole mode. *Water* **11**, 1302 (2019).

66. Lin, L., Chen, C. & Luo, M. Impacts of El Niño–Southern Oscillation on heat waves in the Indochina peninsula. *Atmospheric Science Letters* **19**, e856 (2018).

67. Räsänen, T. A., Lindgren, V., Guillaume, J. H. A., Buckley, B. M. & Kummu, M. On the spatial and temporal variability of ENSO precipitation and drought teleconnection in mainland Southeast Asia. *Climate of the Past* **12**, 1889–1905 (2016).

68. Yin, S. Biomass burning spatiotemporal variations over South and Southeast Asia. *Environment International* **145**, 106153 (2020).

69. Page, S. *et al.* Restoration Ecology of Lowland Tropical Peatlands in Southeast Asia: Current Knowledge and Future Research Directions. *Ecosystems* **12**, 888–905 (2009).

70. Reddington, C. L. *et al.* Contribution of vegetation and peat fires to particulate air pollution in Southeast Asia. *Environmental Research Letters* **9**, 094006 (2014).

71. Tacconi, L. Preventing fires and haze in Southeast Asia. *Nature Climate Change* **6**, 640–643 (2016).

72. Pan, X., Chin, M., Ichoku, C. M. & Field, R. D. Connecting Indonesian Fires and Drought With the Type of El Niño and Phase of the Indian Ocean Dipole During 1979–2016. *Journal of Geophysical Research: Atmospheres* **123**, 7974–7988 (2018).

73. Lestari, D. O., Sutriyono, E., Sabaruddin & Iskandar, I. Severe Drought Event in Indonesia Following 2015/16 El Niño/positive Indian Dipole Events. *Journal of Physics: Conference Series* **1011**, 012040 (2018).

74. Siegert, F., Ruecker, G., Hinrichs, A. & Hoffmann, A. A. Increased damage from fires in logged forests during droughts caused by El Niño. *Nature* **414**, 437–440 (2001).

75. Reid, J. S. *et al.* Multi-scale meteorological conceptual analysis of observed active fire hotspot activity and smoke optical depth in the Maritime Continent. *Atmospheric Chemistry and Physics* **12**, 2117–2147 (2012).

76. Taufik, M. *et al.* Amplification of wildfire area burnt by hydrological drought in the humid tropics. *Nature Climate Change* **7**, 428–431 (2017).

77. Wooster, M. J., Perry, G. L. W. & Zoumas, A. Fire, drought and El Niño relationships on Borneo (Southeast Asia) in the pre-MODIS era (1980–2000). *Biogeosciences* **9**, 317–340 (2012).

78. Fang, K. *et al.* ENSO modulates wildfire activity in China. *Nature Communications* **12**, 1764 (2021).

79. Resco de Dios, V., Yao, Y., Cunill Camprubí, À. & Boer, M. M. Fire activity as measured by burned area reveals weak effects of ENSO in China. *Nat Commun* **13**, 4316 (2022).

80. Chan, J. C. L. & Zhou, W. PDO, ENSO and the early summer monsoon rainfall over south China. *Geophysical Research Letters* **32**, (2005).

81. Li, S. & Bates, G. T. Influence of the Atlantic Multidecadal Oscillation on the winter climate of East China. *Advances in Atmospheric Sciences* **24**, 126–135 (2007).

82. Wu, Z., Li, J., Wang, B. & Liu, X. Can the Southern Hemisphere annular mode affect China winter monsoon? *Journal of Geophysical Research: Atmospheres* **114**, (2009).

83. Yao, Q. *et al.* Pacific-Atlantic Ocean influence on wildfires in northeast China (1774 to 2010). *Geophysical Research Letters* **44**, 1025–1033 (2017).

84. Rogers, J. & McHugh, M. On the separability of the North Atlantic oscillation and Arctic oscillation. *Climate Dynamics* **19**, 599–608 (2002).

85. Hamouda, M. E., Pasquero, C. & Tziperman, E. Decoupling of the Arctic Oscillation and North Atlantic Oscillation in a warmer climate. *Nature Climate Change* **11**, 137–142 (2021).

86. Kim, J.-S., Kug, J.-S., Jeong, S.-J., Park, H. & Schaepman-Strub, G. Extensive fires in southeastern Siberian permafrost linked to preceding Arctic Oscillation. *Science Advances* **6**, eaax3308.

87. Balzter, H. *et al.* Impact of the Arctic Oscillation pattern on interannual forest fire variability in Central Siberia. *Geophysical Research Letters* **32**, (2005).

88. Balzter, H. *et al.* Coupling of Vegetation Growing Season Anomalies and Fire Activity with Hemispheric and Regional-Scale Climate Patterns in Central and East Siberia. *Journal of Climate* **20**, 3713–3729 (2007).

89. Li, J., Fan, K. & Xu, Z. Links between the late wintertime North Atlantic Oscillation and springtime vegetation growth over Eurasia. *Climate Dynamics* **46**, 987–1000 (2016).

90. Lim, Y.-K. The East Atlantic/West Russia (EA/WR) teleconnection in the North Atlantic: climate impact and relation to Rossby wave propagation. *Climate Dynamics* **44**, 3211–3222 (2015).

91. Freitag, M. *et al.* Post‐soviet shifts in grazing and fire regimes changed the functional plant community composition on the Eurasian steppe. *Global Change Biology* **27**, 388–401 (2021).

92. Dubinin, M., Luschekina, A. & Radeloff, V. C. Climate, livestock, and vegetation: what drives fire increase in the arid ecosystems of southern Russia? *Ecosystems* **14**, 547–562 (2011).

93. Xu, Y., Lin, Z. & Wu, C. Spatiotemporal variation of the burned area and its relationship with climatic factors in Central Kazakhstan. *Remote Sensing* **13**, 313 (2021).

94. Jiao, C. *et al.* The impact of teleconnections on the temporal dynamics in aboveground net primary productivity of the Mongolian Plateau grasslands. *International Journal of Climatology* **41**, 6541–6555 (2021).

95. San-Miguel-Ayanz, J. *et al.* Comprehensive monitoring of wildfires in europe: the European Forest Fire Information System (EFFIS). in *Approaches to Managing Disaster - Assessing Hazards, Emergencies and Disaster Impacts* (ed. Tiefenbacher, J.) 87–105 (2012).

96. Brönnimann, S., Xoplaki, E., Casty, C., Pauling, A. & Luterbacher, J. ENSO influence on Europe during the last centuries. *Climate Dynamics* **28**, 181–197 (2006).

97. Mariotti, A., Zeng, N. & Lau, K.-M. Euro-Mediterranean rainfall and ENSO—a seasonally varying relationship. *Geophysical Research Letters* **29**, 59–1 (2002).

98. Hurrell, J. W. Decadal Trends in the North Atlantic Oscillation: Regional Temperatures and Precipitation. *Science* **269**, 676–679 (1995).

99. Trigo, R., Osborn, T. & Corte-Real, J. The {North} {Atlantic} {Oscillation} influence on {Europe}: climate impacts and associated physical mechanisms. *Clim. Res.* **20**, 9–17 (2002).

100. Mellado-Cano, J., Barriopedro, D., García-Herrera, R., Trigo, R. M. & Hernández, A. Examining the North Atlantic Oscillation, East Atlantic Pattern, and Jet Variability since 1685. *Journal of Climate* **32**, 6285–6298 (2019).

101. Ionita, M. The Impact of the East Atlantic/Western Russia Pattern on the Hydroclimatology of Europe from Mid-Winter to Late Spring. *Climate* **2**, 296–309 (2014).

102. Bonan, G. B., Chapin, F. S. & Thompson, S. L. Boreal forest and tundra ecosystems as components of the climate system. *Climatic Change* **29**, 145–167 (1995).

103. Turetsky, M. R. *et al.* Global vulnerability of peatlands to fire and carbon loss. *Nature Geoscience* **8**, 11–14 (2015).

104. Drobyshev, I. *et al.* Reconstruction of a regional drought index in southern Sweden since AD 1750. *The Holocene* **21**, 667–679 (2011).

105. Li, M. *et al.* Collaborative impact of the NAO and atmospheric blocking on European heatwaves, with a focus on the hot summer of 2018. *Environmental Research Letters* **15**, 114003 (2020).

106. Abram, N. J. *et al.* Connections of climate change and variability to large and extreme forest fires in southeast Australia. *Communications Earth & Environment* **2**, 1–17 (2021).

107. Boer, M. M., Resco de Dios, V. & Bradstock, R. A. Unprecedented burn area of Australian mega forest fires. *Nature Climate Change* **10**, 171–172 (2020).

108. McGregor, S. *et al.* Recent Walker circulation strengthening and Pacific cooling amplified by Atlantic warming. *Nature Climate Change* **4**, 888–892 (2014).

109. Xue, J. *et al.* Decadal-scale teleconnection between South Atlantic SST and southeast Australia surface air temperature in austral summer. *Climate Dynamics* **50**, 2687–2703 (2018).

110. Wei, W., Yan, Z. & Li, Z. Influence of Pacific Decadal Oscillation on global precipitation extremes. *Environmental Research Letters* **16**, 044031 (2021).

111. Bureau of Meteorology (Australian Government). The Indian Ocean Dipole (IOD). *http://www.bom.gov.au/climate/enso/history/ln-2010-12/IOD-what.shtml* (2022).

112. Nagaraju, C., Ashok, K., Balakrishnan Nair, T. M., Guan, Z. & Cai, W. Potential influence of the Atlantic Multi-decadal Oscillation in modulating the biennial relationship between Indian and Australian summer monsoons. *International Journal of Climatology* **38**, 5220–5230 (2018).

113. Power, S., Casey, T., Folland, C., Colman, A. & Mehta, V. Inter-decadal modulation of the impact of ENSO on Australia. *Climate Dynamics* **15**, 319–324 (1999).

114. Mariani, M., Veblen, T. T. & Williamson, G. J. Climate Change Amplifications of Climate-Fire Teleconnections in the Southern Climate Change Ampli fi cations of Climate-Fire Teleconnections in the Southern Hemisphere. *Geophysical Research Letters* **45**, (2018).
